# Differential Mobility Spectrometry of Ketones in Air at Extreme Levels of Moisture

**DOI:** 10.1038/s41598-019-41485-7

**Published:** 2019-04-03

**Authors:** Z. Safaei, G. A. Eiceman, J. Puton, J. A. Stone, M. Nasirikheirabadi, O. Anttalainen, M. Sillanpää

**Affiliations:** 1 Department of Green Chemistry, LUT University, Sammonkatu 12, FI-50130 Mikkeli, Finland; 2 Department of Chemistry and Biochemistry, 1175 North Horseshoe Drive, New Mexico State University, Las Cruces, NM 88003 USA; 3 Institute of Chemistry, Military University of Technology, Kaliskiego 2, Warsaw, Poland; 4 Department of Chemistry, Queens University, Kingston, Ont. K7L 4J1 Canada; 5 Environics Oy, Sammonkatu 12, FI-50130 Mikkeli, Finland

## Abstract

The performance of a differential mobility spectrometer was characterized at ambient pressure and ten values of water vapor concentration, from 1.0 × 10^2^ to 1.7 × 10^4^ ppm using a homologous series of seven ketones from acetone to 2-dodecanone. Dispersion plots at 30 °C with separation fields from 35 to 123 Td exhibited increased alpha functions for the hydrated proton, protonated monomers, and proton bound dimers with increased moisture levels. Increases in the level of moisture were accompanied by decreased quantitative response with progressive suppression in the formation of the proton bound dimer first and then protonated monomer. Product ions for 2-octanone at 7 ppb were not observed above a moisture level of 4.0 × 10^3^ ppm, establishing a limit for observation of analyte ion formation. The observation limit increased from 1.1 × 10^3^ ppm for acetone to 5.7 × 10^3^ ppm for 2-dodecanone. These findings demonstrate that ketones can be determined with a differential mobility spectrometry (DMS) analyzer near room temperature in the presence of elevated levels of moisture expected with the use of membrane inlets or headspace sampling of surface or ground waters. Moisture levels entering this DMS analyzer employed as an environmental monitor should be kept at 1.0 × 10^3^ ppm or below and quantitative studies for individual ketones should be made at a fixed moisture level.

## Introduction

Differential mobility spectrometry (DMS) is an embodiment of ion mobility spectrometry (IMS) where ion characterization and separation occur as a result of the non-linear dependence of ion velocity on the intensity of an electric field. In DMS instruments, ions are carried by gas flow through a gap formed between two parallel plates. The electric field inside the gap, which is perpendicular to plates, is generated by voltage of an asymmetric waveform^1,2^. The maximum amplitude of the waveform is called the separation voltage (SV). Differences in mobility coefficients between the two extremes of the asymmetric oscillating electric field cause off-axis displacement and loss of ions during passage through the gap. Ions can be restored to the gap center and passed to a detector when a weak electric field, the compensation field (CF), is superimposed on the oscillating field. Analytical information from DMS instruments is contained in the differential mobility spectrum, the dependence of ion current on CF. The plot of ion abundance as a function of separation field (SF) and CF is called a dispersion plot.

The dependence of the ion mobility coefficient *K(E/N)* on the electric field *E* can be described by the formula:

1 $$K(\frac{E}{N})={K}_{0}(1+\alpha (\frac{E}{N}))$$

Where *N* is the number density of the gas (number of molecules per cubic centimeter), *K*_0_ is the mobility measured under low field conditions and α is a function characterizing the dependence of mobility on electric field^3^. Since mobility coefficients are sensitive to gas temperature^4^, moisture^5,6^, pressure^7^ and gas composition^8^, these parameters should be controlled for the stable and reliable performance of DMS and other mobility analyzers. The simplicity of design and fabrication of DMS analyzer have encouraged applications in chemical measurements from monitoring of air quality on the International Space Station^9^ to pre-filtering ions prior to mass spectrometry^10^. Hand held DMS analyzers have been developed for military use to detect chemical warfare agents, demonstrating an embodiment of DMS for demanding on-site measurements.

In chemical analysis performed with DMS, substances are ionized through reactions with precursor ions formed in ionization regions at ambient pressure often, in purified air or nitrogen^11^. In positive polarity, ionization of a substance (M) occurs through displacement of water from hydrated protons forming relatively simple product ions, typically a protonated monomer MH^+^(H_2_O)_n_ or a proton bound dimer M_2_H^+^(H_2_O)_n_. Such reactions are known from studies using chemical ionization mass spectrometry (CI-MS), atmospheric pressure ionization MS, and IMS-MS with moisture content in supporting gas atmospheres below 1 × 10^2^ ppm. Response to a broad range of substances is possible and can be extended using a range different ionization sources including radioisotopes, photoionization^12,13^ and gas discharge^14^. Simple spectral patterns from such ion sources enable valuable analytical performance with comparatively low resolving power technology. The separation of ion peaks in DMS for improved analytical performance in some instances can be enhanced by adding into the gas atmosphere up to 1.0 × 10^3^ ppm of small polar molecules^15–17^. Such substances, called vapor modifiers, increase the differences in mobility coefficients at asymmetric field extremes, resulting in increased separation of ion peaks in the compensation field. Moisture can be considered a vapor modifier in DMS.

Complimented by advantages of simplicity, size, and low cost, the use of DMS analyzers for monitoring aqueous environments is also supported by prior success of drift tube IMS instruments for monitoring ammonia in surface waters^18^, haloacetic acids in drinking water^19^, thiotetronic acids in groundwater^20^ and in general environmental measurements^21^. Environmental monitoring of water using DMS analyzers is nonetheless uncommon, although some successes have been reported^22,23^. Interfaces to aqueous samples such as headspace samplers or membrane inlets^24^ and likely will introduce moisture into the DMS.

A central concern in this research program is the influence of moisture on DMS response and performance, necessitating quantitative descriptions and managed parameters. Previous studies in MS, DMS, and drift tube IMS have shown that increases in moisture suppress response, though this can be reversed with increased temperature^6,25–28^ Particularly, the recent work of Borsdorf, *et al*.^28^ who systematically evaluated the effect of increased moisture on the reactant ion peak in an ion mobility spectrometer at 80 °C and up to 2.0 × 10^3^ ppm moisture. Quantitative response with elevated moisture levels was diminished by >90% with aromatic compounds and >70% with ketones. Response to high proton affinity aniline, 2-methyl aniline and substituted anilines was diminished by <10%. In our DMS instrument, temperature was limited to 40 °C because the analyzer stage is fabricated from printed circuit boards.

As part of a development program leading to a field-deployed instrument for water monitoring, understandings of the quantitative influence of moisture on response and performance in DMS are necessary. Ketones were chosen to benchmark performance over a wide range of moisture levels with some assurance of favorable ionization chemistry from the considerably higher proton affinity of ketones compared with that of water. The embodiment of DMS chosen for water monitoring in Finland is a prototype instrument from Environics Oy with a capacity to operate at room temperature with high levels of moisture and could be suitable for directly monitoring aquatic enviroments. Findings in this study will inform designs of the next stage toward development of an inlet or interface to aqueous samples and the levels of moisture suitable for response to volatile organic compounds. Results on the influence of moisture on the ionization of ketones may be useful also in understanding DMS response to other analytes using atmospheric pressure ionization in the positive mode.

The main aim of our project was to develop the AIMS2-DMS detector for environmental pollutant monitoring. The aspiration module (AIMS) part of the equipment was not investigated in our study, however its features are promising for selected applications. An advantage of DMS over drift tube IMS is favorable performance with samples in humid air. In DMS, the changes in peak position or dispersion plots with differing moisture content might provide quantitative information about moisture levels in a sample. Since DMS analyzers are simpler and smaller than drift-tube IMS analyzers, they are highly suitable for portable applications.

## Experimental

### Instrumentation

#### Differential Mobility Analyzer

**Figure 1:**
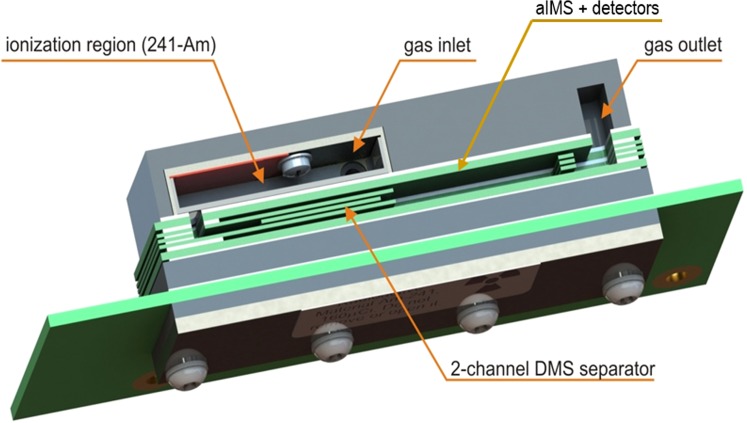
Graphic of analyzer region of differential mobility spectrometer (top frame) showing location of ion source and two mobility stages based on differential mobility spectrometry (DMS) and aspirator IMS region including three detectors. Schematic in bottom frame shows side shows principle of two stage analyzer. In these studies, only performance of the first (DMS) stage was explored. The analyzer was equipped with an inline pressure sensor.

**Figure 2:**
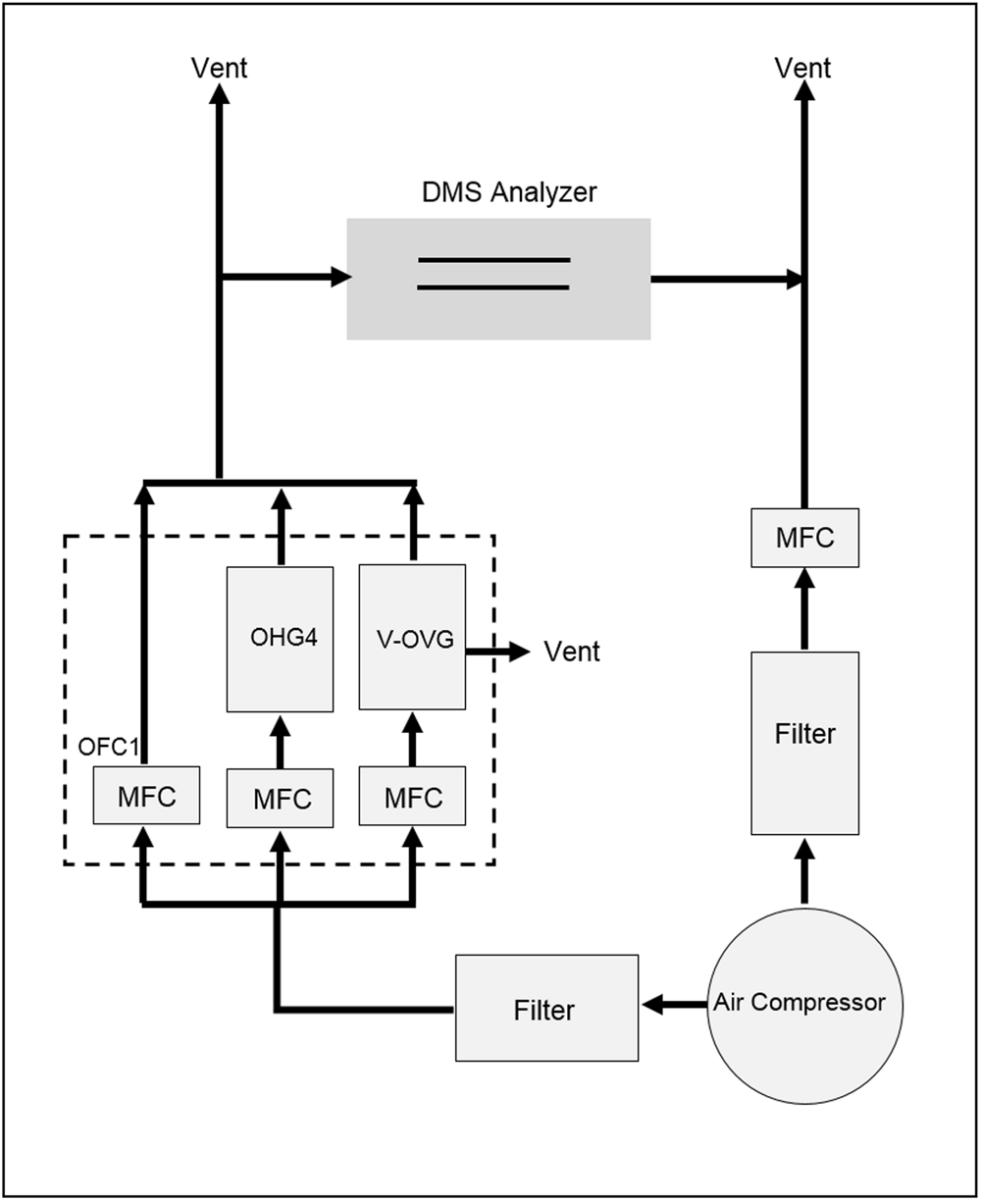
Schematic of sample vapor and gas flows including DMS analyzer, mass flow controllers (MFC), and vapor generator comprised of dilution flow (OFC1), humidifier (OHG4), and permeation chamber (V-OVG). Flow though the DMS analyzer was controlled using the Venturi effect. Excess flows, shown as Vent, entered a fume hood.

A model AIMS2-DMS analyser (Environics OY, Mikkeli, Finland) consists of an aspiration ion mobility spectrometer (AIMS) and a two-channel DMS detector as shown in the analyzer structure (Fig. 1). The ionization source is ^241^Am with 5.92 MBq activity. A DMS drift region consists of two parallel rectangular electrodes 6 mm wide × 14 mm long, separated by a 0.25 mm gap defined by a PTFE spacer. The DMS stage was provided an asymmmetric waveform with duty cycle of 5% and frequency of 250 kHz. The DMS was operated at 30 °C and ambient pressure monitored by a pressure sensor in-line with the analyzer. Sample flow was drawn into the DMS analyzer at a rate of 2.8 L min^−1^ by the Venturi effect (Fig. 2). Measurements were made using the SV from 200 V to 650 V in 45 steps and the CV was scanned from −2 V to +12 V in 400 increments. Amplifier dual time at each voltage combination was 150 ms. For each experiment, a disperson curve was determined. Details of the AIMS2-DMS analyzer including sensitivity and resolving power have been described^28^.

#### Vapor Generator

Four ml or more of a chemical were placed in a permeation tube, built from a stainless steel tube capped with a polydimethylsiloxane membrane 3 mm thick × 1 mm diameter, and held at 30 °C for a minimum of one week before use. The permeation rate was determined gravimetrically over two or more weeks. Flow from the permeation tube (in the V-OVG chamber) was introduced into the DMS analyzer as shown in Fig. 2. The vapor generator (Gen-Sys, Owlstone Ltd., Cambridge, UK) was equipped also with a model OHG-4 humidity generator and model OFC-1 (dilution flow) flow controller to allow humidity and vapor concentration to be accurately adjusted. The accuracy of adjustment for flow rate is 0.1 ml min^−1^ and of temperature for V-OVG is 0.1 °C. The generator provided air, purified through an activated carbon filter (Carbon Capsule Filter, Pall Laboratory, VWR) and a mixed bed of 4 A, 5 A, and 13X molecular sieve. The air supply was an Atlas Copco SF 6 FF oil free compressor. Vapor concentration was adjusted to 7 ppb using generator flow, split flow, and dilution flow containing known moisture level from OHG-4. Gas pressure provided to the vapor generator was maintained at 40 psig. Sample flow was at a rate greater than that sampled by the DMS analyzer with the excess vented into a fume hood.

### Chemicals

Seven ketones of analytical standard grade (Sigma-Aldrich Corp., St. Louis, MO), acetone, 2-butanone, 2-hexanone, 2-octanone, 2-nonanone, 2-decanone, and 2-dodecanone represent a homologous series. Deionized water for the humidity generator was from an Arium® mini purifier (Ultrapure Water System, Sartorius, Germany).

### Procedures

#### Studies of Response with Different Moisture Levels

Control measurements for the reactant ion were made without analyte from 1.0 × 10^2^ ppm to 1.71 × 10^4^ ppm moisture in 37 steps. Measurements were then repeated for the individual ketones at 7 ppb over the same moisture levels.

#### Data Analysis

Data sets were processed using MATLAB (The MathWorks Inc., Natick, MA) to determine CV for peak positions and peak intensities at each SV and stored as a data matrix. Electric field intensities (SF and CF) were calculated on the basis of corresponding voltages (SV and CV) using gap width (0.25 mm). Values of electric field were normalized to number density (*N*) as *E*/*N* with units of Townsends (1 Td = 10^−21^ V m^−2^). Barometric pressure inside the DMS separator was nominally 94.9 kPa over the course of these measurements, which gives at 30 °C the number density of 2.27 × 10^25^ molecules m^−3^. Thus, a value for SV of 500 V produced a field of 2.0 × 10^4^ V cm^−1^, and E/N = 88 Td.

## Results and Discussion

### DMS spectra for the reactant ion for a wide range of moisture

**Figure 3:**
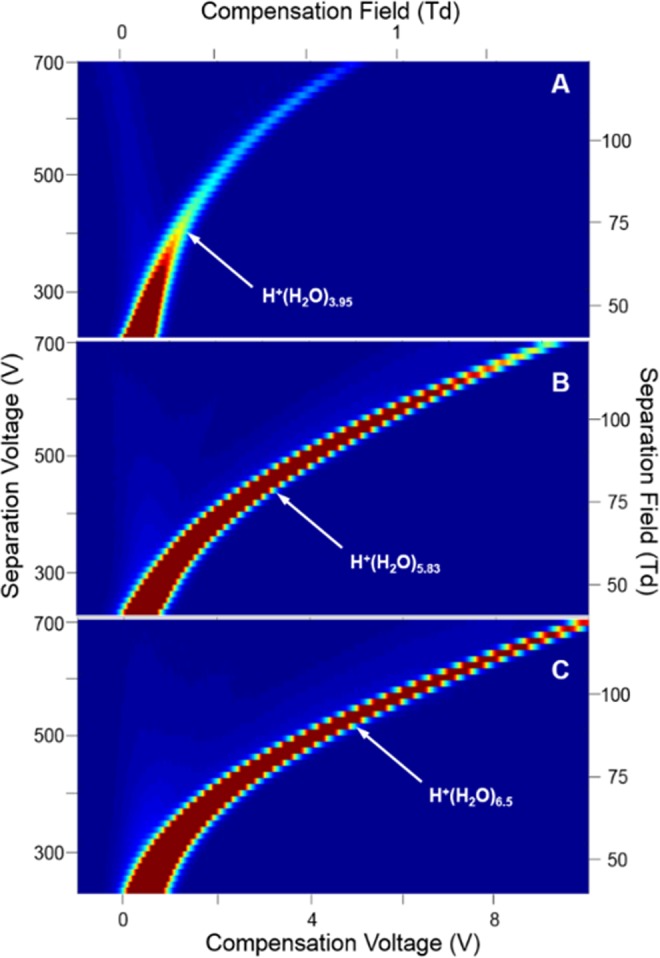
Dispersion plots for the reactant ion H^+^(H_2_O)_n_ at moisture levels of (**A**) 1.0 × 10^2^, (**B**) 6.00 × 10^3^, and (**C**) 1.71 × 10^4^ ppm in purified air; average values at these levels for n are 3.95, 5.83, and 6.50, respectively.

Mobility spectra obtained in the absence of analyte were repeatable showing a single peak, due to the reactant ion, the hydrated proton, H^+^(H_2_O)_n_^29,30^. The field dependence of this peak is seen in the characteristic DMS dispersion plot in Fig. 3A for 1.0 × 10^2^ ppm moisture. The shape arises from the difference in mobility coefficients between the hydration levels of H^+^(H_2_O)_n_ at extremes of the separation field intensity. The alpha function (Eq. 1) from this plot is relatively shallow for a 13 mm long analyzer region yet consistent with the 5% duty cycle of the asymmetric waveform, against 30% for similar DMS analyzers^3,31^.

A moisture level of 1.0 × 10^2^ ppm was the minimum employed for the study of ketones since lower levels were deemed unrealistic for in-field monitors of aqueous media with headspace samplers or membrane inlets. When the moisture level was increased to 6.00 × 10^3^ ppm (Fig. 3B) and 1.71 × 10^4^ ppm (Fig. 3C) the alpha function increased with greatest change between 1.0 × 10^2^ and 6.00 × 10^3^ ppm and least change between 6.00 × 10^3^ and 1.71 × 10^4^ ppm. Water functions as a vapor modifier expanding the range of compensation voltage and is a favorable, welcome influence, mindful of the inevitable participation of moisture from sampling surface or ground waters^32^. Significantly, no fouling or dysfunction occurred with this analyzer despite these high moisture levels.

**Figure 4:**
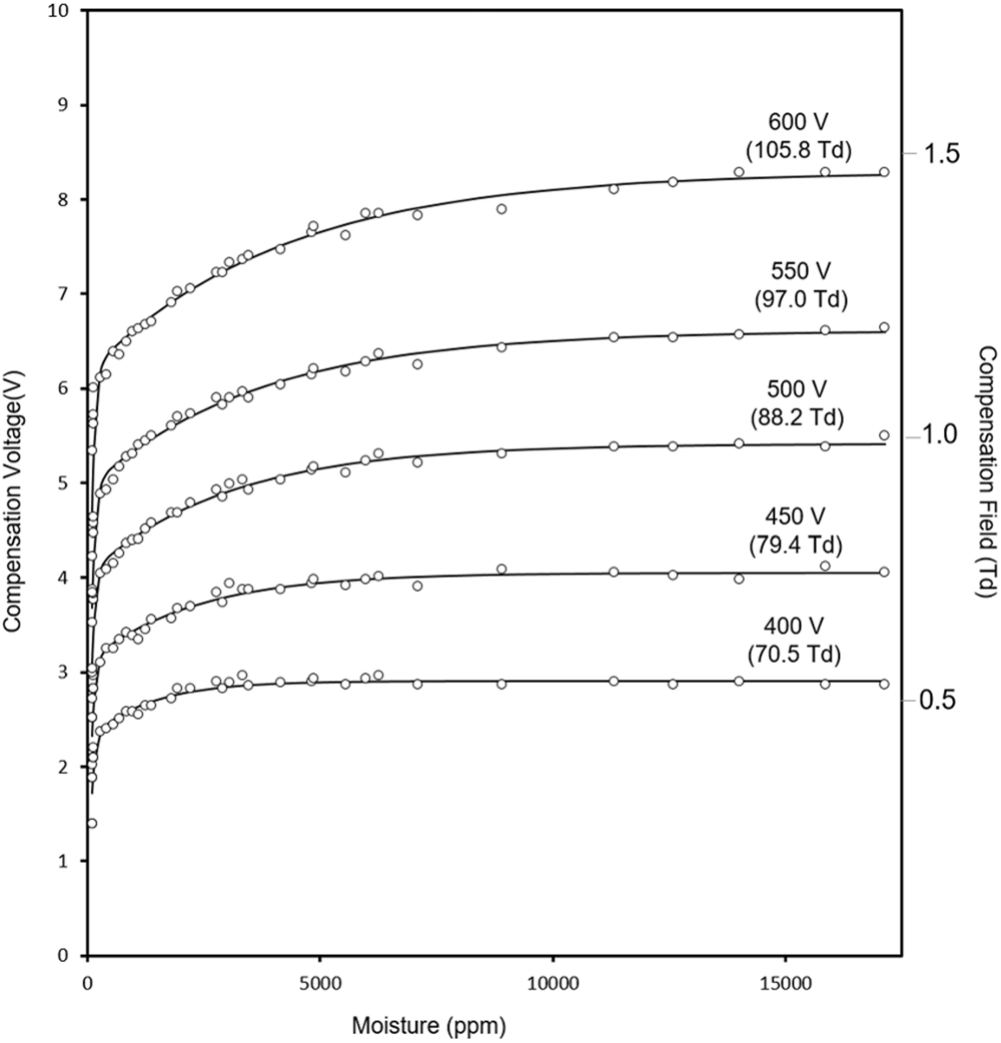
Compensation voltage (and corresponding compensation field) for the hydrated proton at moisture levels from 1.0 × 10^2^ to 1.71 × 10^4^ ppm at five values of separation voltages as shown above each curve with corresponding separation field in parentheses.

**Figure 5:**
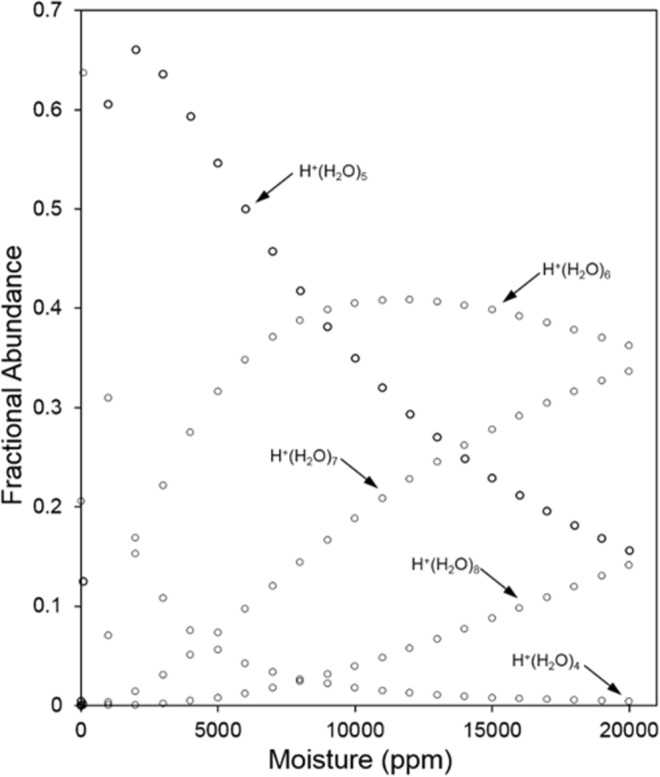
Plots for the distribution of species H^+^(H_2_O)_n_ at 30 °C at equilibrium with moisture levels from 1.0 × 10^2^ to 2.00 × 10^4^ ppm, calculated from literature data^29^.

Measured relationships between the moisture level and CV are shown in Fig. 4 over the range 1.00 × 10^3^ ppm to 1.71 × 10^4^ ppm at five settings of SV^33^. The shapes of all plots follow a common pattern with a large change between 1.0 × 10^2^ and 1.00 × 10^3^ ppm followed by a gradual decrease in slope. Since CV is a measure of the difference of mobility values for ions at field extremes, this response above 4.00 × 10^3^ ppm suggests the impact of the moisture level on alpha functions reaches a limit. The cause for this is attributed to the differing level of proton hydration. As is shown in Fig. 5, calculated fractional abundances of H^+^(H_2_O)_n_ ions for n = 3, 4 and 5 at 30 °C and 100 ppm H_2_O are 0.245, 0.635, and 0.135, respectively giving an average value of n of 3.95, which corresponds to an average ion mass of 72 Da. At 1.00 × 10^3^ ppm H_2_O, the average value for n is 4.52 and the average ion mass is 82 Da. Over this range in moisture levels, heat capacities of these ions and dehydration during the high field extreme of SF should be comparable. Increases in mobility differences (and thus CV as suggested in Fig. 4) arise from differences in hydration levels during the low extreme of SF.

As the level of moisture is increased above 1.00 × 10^3^ ppm, the dehydration step should decrease due to increased heat capacity of more heavily hydrated H^+^(H_2_O)_n_ where average values for n are 5.34 at 5.00 × 10^3^ ppm, 5.91 at 1.00 × 10^4^ ppm, 6.27 at 1.50 × 10^4^ ppm, and 6.50 at 1.71 × 10^4^ ppm. This corresponds to average ion masses (Da) of 96.76, 107.38, 113.86, and 118.0. An increase in hydration of the ion at the low field extreme of SF has occurred in Fig. 4 yet limited dehydration at the high field extreme is suggested by the slopes. Nonetheless, a shallow, yet discernable, slope for SV = 600 V for example is evidence that SF, even with 5% duty cycle, is sufficient to heat the ions, producing a change in ΔK and hence CV, even with extreme levels of moisture.

Values for peak full width at half-maximum (FWHM) which are established initially by the distance of the gap between the plates in the separation region (for SF = 0) and decreased with increased SV from 400 V to 600 V at all levels of moisture as shown for four levels of moisture in Table 1. FWHM also decreased with decreased moisture. Both trends can be understood on the basis of the simple model for the DMS analysers^27^.

**Table 1:** Full width at half maximum (FWHM in V) for reactant ion peak at four levels of moisture and six values of separation voltage (field).

H_2_O (ppm)	1.0 × 10^2^	1.00 × 10^3^	4.00 × 10^3^	1.71 × 10^4^
Separation Voltage (V) (Separation Field, Td)				
400 (70.5)	0.61	0.66	0.70	0.72
450 (79.4)	0.60	0.65	0.67	0.70
500 (88.2)	0.56	0.63	0.66	0.68
550 (97)	0.54	0.62	0.64	0.66
600 (105.8)	0.48	0.60	0.63	0.65
650 (114.6)	0.43	0.58	0.61	0.63

**Figure 6:**
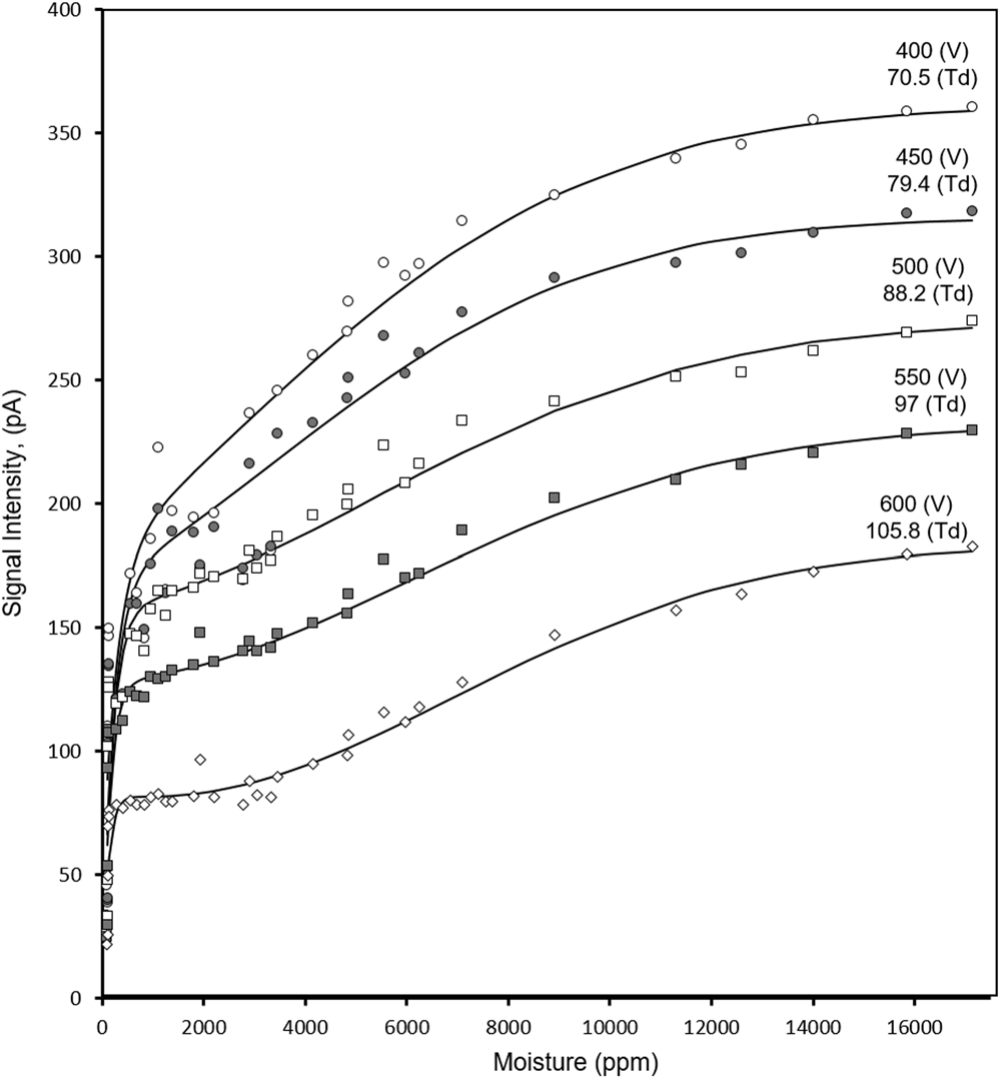
Peak intensity for H^+^(H_2_O)_n_ at five Separation Voltages (V) or Separation Fields (Td) at moisture levels from 1.0 × 10^2^ to 1.71 × 10^4^ ppm.

A secondary effect from increased moisture levels was increased intensity for the hydrated proton peak as shown in Fig. 6. The slope of these plots exhibit an initial rapid rise from 1.0 × 10^2^ ppm to 1.00 × 10^3^ ppm and then an increasingly smaller rise to 1.71 × 10^4^ ppm. Over the entire moisture span, the average mass of H^+^(H_2_O)_n_ increases from 75.05 Da to 123.5 Da as the average value for n increases from 3.95 to 6.5. Since ion transmission efficiency in planar DMS analyzers is mass-dependent the trends in peak intensity observed in Fig. 6 can be attributed to changes in the mass of the hydrated proton. The effective gap for H^+^(H_2_O)_n_ increases with moisture level and ion transmission is increased (Table 1) at each SF.

### DMS response for example analyte (2-octanone) over the range of moisture

**Figure 7:**
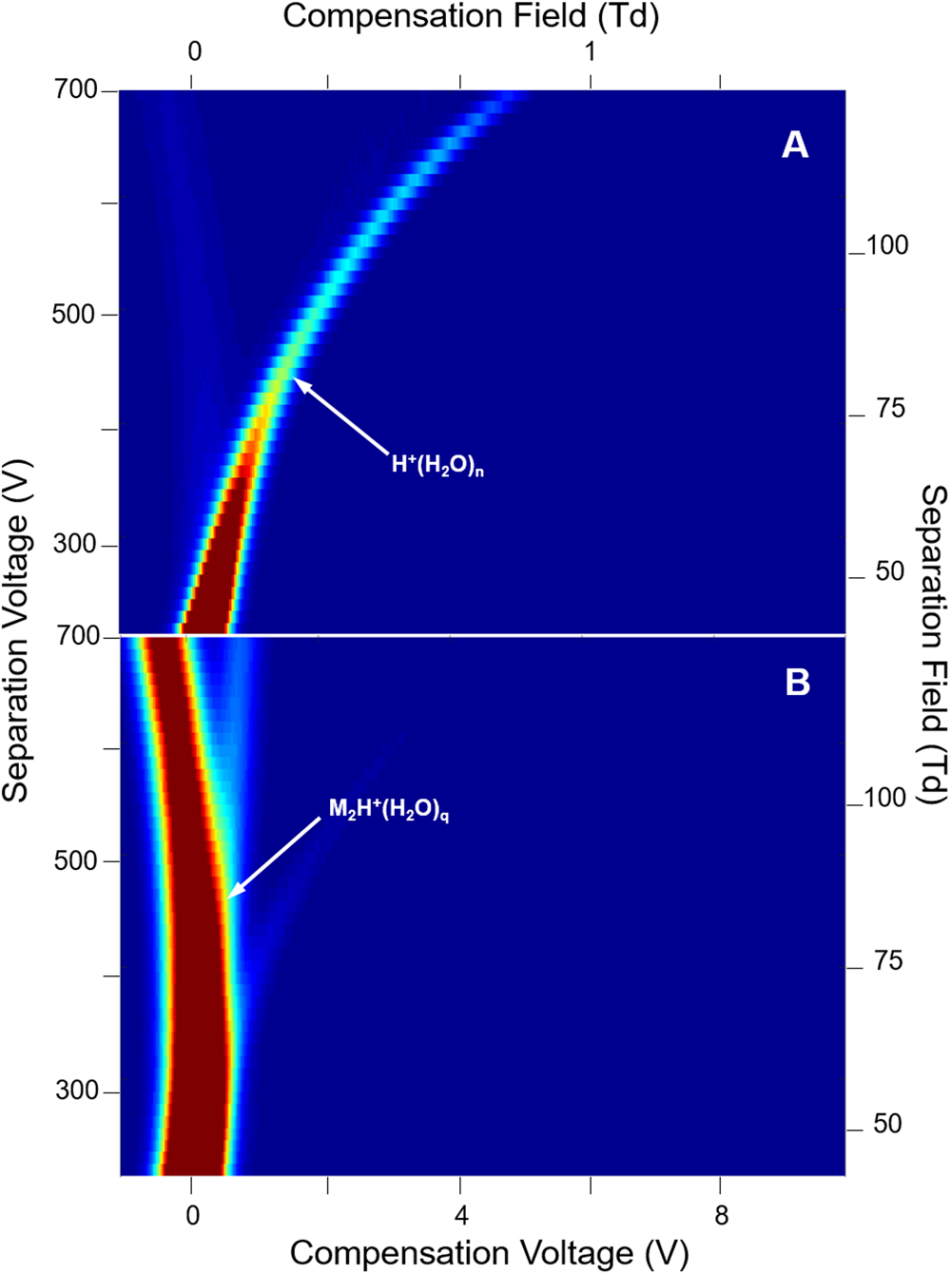
Dispersion Plots for (**A**) the reactant ion and for (**B**) product ions from 2-octanone at 7 ppb in air at a moisture level of 1.0 × 10^2^ ppm. Ions with positive dependence of SF on ΔK (positive alpha function) trend toward positive CV, i.e., CF in this analyzer. Average n = 3.95.

**Figure 8:**
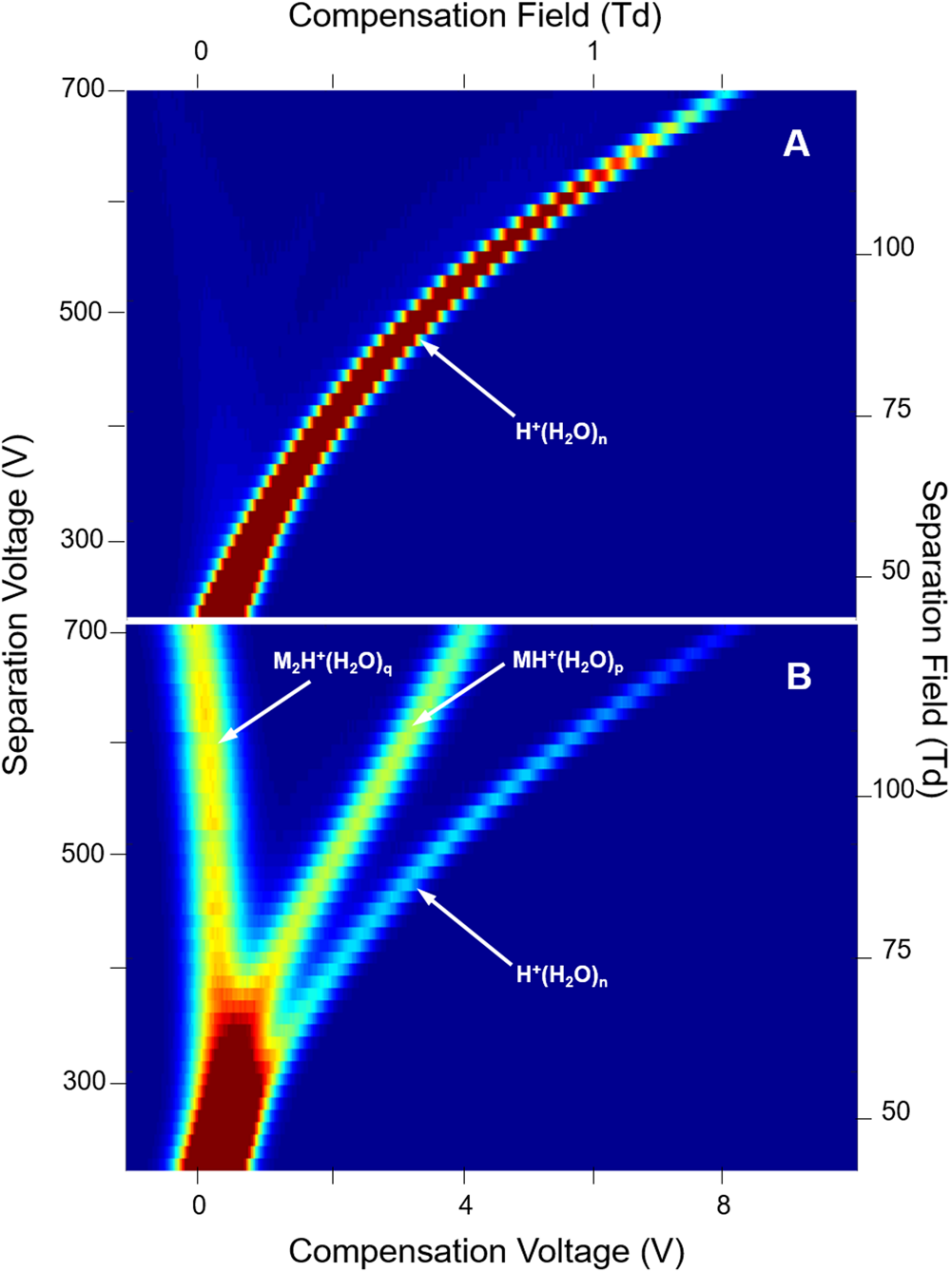
Dispersion Plots for (**A**) the reactant ion and (**B**) for product ions from 2-octanone at 7 ppb in air at a moisture level of 1.40 × 10^3^ ppm. Average n = 4.98.

**Figure 9:**
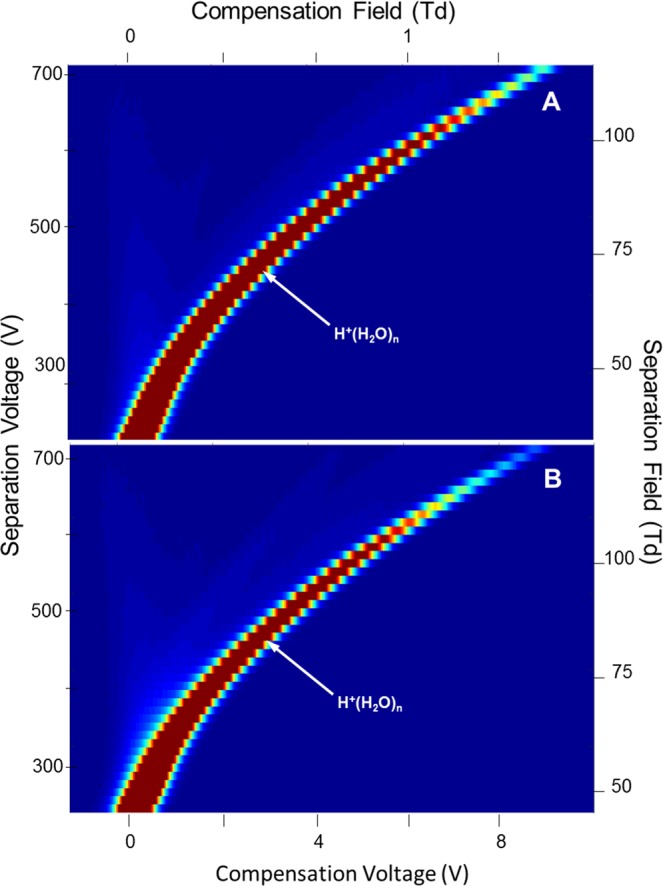
Dispersion Plots for (**A**) the reactant ion and (**B**) for product ions from 2-octanone at 7 ppb in air at a moisture level of 4.00 × 10^3^ ppm. Average n = 5.28.

While elevated moisture levels from sampling ground or surface waters may have favorable benefits in DMS (see above), moisture can also affect the chemistry of ion formation. The typical behavior of ketone ions with increased levels of moisture is shown in dispersion plots for 2-octanone (7 ppb) in Figs 7–9. In each figure, the dispersion plot for the reactant ion peak is shown as a reference of instrument response without ketone vapor. At a moisture level of 1.0 × 10^2^ ppm, the reactant ion (Fig. 7, top frame) is replaced in the presence of 2-octanone vapor with a proton bound dimer (Fig. 7, bottom frame) that is characterized by a slight trend with increased SF to a more negative CF, that is a negative alpha function. An initial collision complex [MH^+^(H_2_O)_n_]* formed between M and the hydrated proton is stabilized by collision with ambient gas molecules G (Eq. 2). The hydration shell is changed, probably by loss of one or more water molecules, the number denoted by x in the equation:

2 $${H}^{+}{({H}_{2}O)}_{n}+M\to {[M{H}^{+}{({H}_{2}O)}_{n}]}^{\ast }\mathop{\longrightarrow }\limits^{G}M{H}^{+}{({H}_{2}O)}_{(n-x)}+x{H}_{2}O$$

MH^+^(H_2_O)_(n−x)_ will change its degree of hydration by coming to equilibrium with water in the atmosphere. We denote the new hydration number by p in Eq. 3 that illustrates the formation of the proton bound dimer by further reaction of MH^+^(H_2_O)_p_, again with probable change in hydration number, again denoted by *x*. Because the hydrogen bond in symmetrical proton-bound dimers is always greater than between non-symmetrical proton-bound dimers, thermodynamic considerations lead to preferential formation of the symmetrical dimers^34,35^. The rate at which symmetrical dimer formation occurs depends in the present case on the degree of solvation of the monomer as per Eq. 2.

3 $$M{H}^{+}{({H}_{2}O)}_{p}+M\to {[{M}_{2}{H}^{+}{({H}_{2}O)}_{P}]}^{\ast }\mathop{\longrightarrow }\limits^{G}{M}_{2}{H}^{+}{({H}_{2}O)}_{(p-x)}+x{H}_{2}O$$

**Figure 10:**
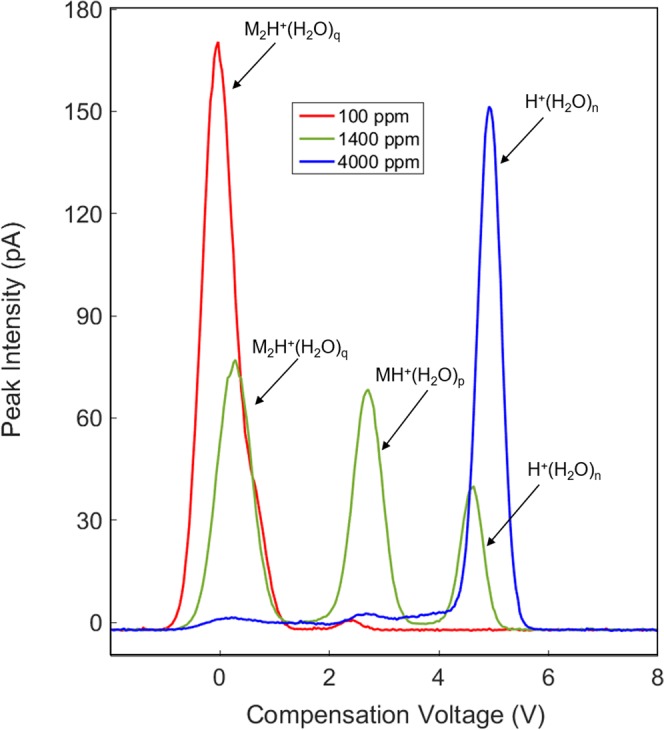
Mobility spectra for reactant ion and 2-octanone at separation voltage of 500 V (88 Td) and moisture levels of 1.0 × 10^2^, 1.40 × 10^3^, and 4.00 × 10^3^ ppm.

**Figure 11:**
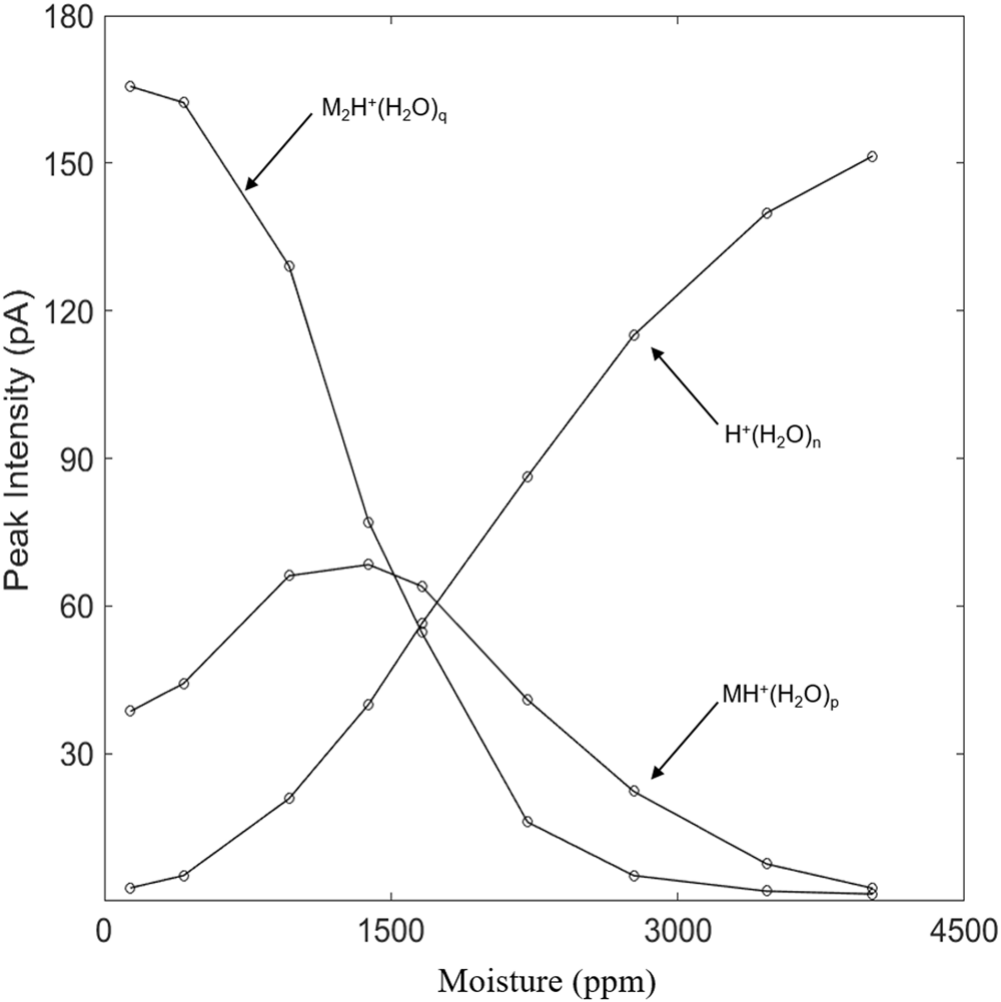
Peak intensity for the reactant ion, protonated monomer, and proton bound dimer of 2-octanone at 7 ppb in air with moisture levels from 1.0 × 10^2^ to 4.00 × 10^3^ ppm at SV = 500 V.

M_2_H^+^(H_2_O)_p–x_ will also come to equilibrium with the atmosphere to give a dimer with average number of water molecules denoted by subscript q (see below). The degrees of ion hydrations, depend on the moisture level and temperature. At a moisture level of 1.40 × 10^3^ ppm (Fig. 8, top frame), the hydrated proton peak exhibits enhanced dispersion in CV and this same ion is evident in the presence of the ketone vapor (Fig. 8, bottom frame). Moreover, the proton bound dimer with intense response at 1.0 × 10^2^ ppm [H_2_O] is decreased in intensity and the protonated monomer with comparable intensity is present. At 4.00 × 10^3^ ppm only the reactant ion peak is present (Fig. 9) and suppression of ionization of 2-octanone is complete. Another view of the changes observed in the spectra obtained with SV = 500 V is shown in Fig. 10 for these same levels of moisture and the quantitative change over the moisture range is shown in Fig. 11. This change in abundance of product ions (while the vapor level of 2-octanone was stable at 7 ppb) can be attributed to changes in the rates of reactions in Eqs 2 and 3 as the hydration level of the participating ions increases with increased moisture levels (see below).

As the moisture level increases, the hydrated proton grows in size (Fig. 5), which leads to an increase in nominal proton affinity of the water cluster (Table 2) that is approximately defined by the enthalpy change for reaction described by Eq. 4.

4 $${H}^{+}{({H}_{2}O)}_{n}\to {H}^{+}+n{H}_{2}O$$

**Table 2:** Proton affinity for H^+^(H_2_O)_n_ for n from 1 to 10.

n	Formula	Proton Affinity (kJ mol^−1^)
1	H^+^(H_2_O)	691
2	H^+^(H_2_O)_2_	827
3	H^+^(H_2_O)_3_	911
4	H^+^(H_2_O)_4_	984
5	H^+^(H_2_O)_5_	1040
6	H^+^(H_2_O)_6_	1090
7	H^+^(H_2_O)_7_	1135
8	H^+^(H_2_O)_8_	1178
9	H^+^(H_2_O)_9_	1219
10	H^+^(H_2_O)_10_	1256

The nominal proton affinities for the clusters with n = 1 to 6 are (kJ mol^−1^) consecutively, 691, 827, 911, 984, 1040, and 1090. In comparison, the proton affinity of 2-octanone, is approximately 850 kJ mol^−1^, an upper limit for listed ketones. (NIST Standard Reference Simulation Website)^36^. Exothermic proton transfer is therefore only possible for H^+^(H_2_O)_n_ with n = 1 or 2. Exothermic proton transfer from H^+^(H_2_O)_n_ to 2-octanone for n ≥ 3 can only occur with concomitant transfer of one or more water molecules as described by Eq. 2.

The collision of 2-octanone with H^+^(H_2_O)_n_ leads to a nascent ion-molecule complex [MH^+^(H_2_O)_n_]^*^ that can either be stabilized by collision, with or without loss of water molecules, or revert to reactants. The reversion will be increasingly favored as n increases because of steric hindrance and some delocalization of charge over the water network. The rate of proton transfer to produce MH^+^(H_2_O)_p_ will decrease and with a sufficiently large n, proton transfer will not occur. The degree of hydration of MH^+^(H_2_O)_p_ also increases with increasing moisture content and the same argument made for MH^+^(H_2_O)_p_ can be made for the decreasing rate of formation of M_2_H^+^(H_2_O)_q_ with increasing moisture content. Figure 11 shows that M_2_H^+^(H_2_O)_q_ decreases monotonically as MH^+^(H_2_O)_p_ first increases, attains a maximum and then decreases while non-reacted H^+^(H_2_O)_n_ increases monotonically. Above 4.00 × 10^3^ ppm moisture no proton transfer is possible for 2-octanone at a concentration of 7 ppb. From Fig. 5 this occurs when H^+^(H_2_O)_5_ is the main reactant ion. The plots in Fig. 11 hold critical importance for analytical measurements of water samples demonstrating that moistures content should be as low as possible for best response Moisture up to 1.00 × 10^3^ ppm is tolerable but with increasing degraded quantitative response. The influence of moisture level on ionization of other ketones should differ based on the trend in proton affinities in the homologous series of ketones (Table 3) where proton affinity ranges from 691 to 840 kJ mol^−1^.

**Table 3:** Proton affinities from Gaussian modeling for a homologous series of ketones and maximal threshold values of humidity above which no ketone detection was possible.

Ketone	Proton Affinity (kJ mol^−1^)	H_2_O (ppm)
Acetone	810	1.10 × 10^3^
2-Butanone	820	1.25 × 10^3^
2-Hexanone	826	3.60 × 10^3^
2-Octanone	835	4.00 × 10^3^
2-Noanone	837	4.20 × 10^3^
2-Decanone	838	5.40 × 10^3^
2-Dodecanone	840	5.70 × 10^3^

### DMS analysis of a homologous series of ketones

**Figure 12:**
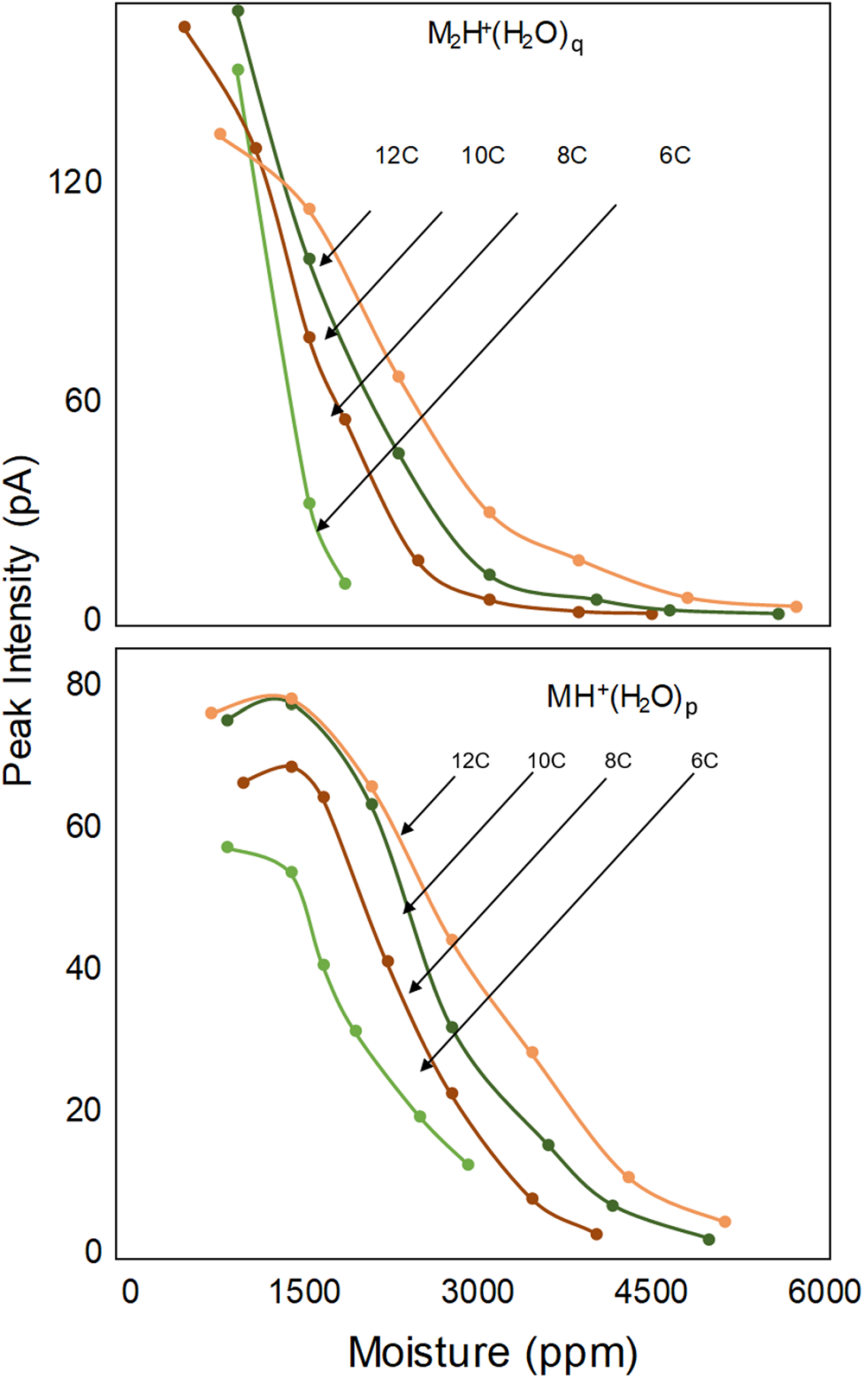
Peak intensity for protonated monomer and proton bound dimer of a homologous series of ketones (C for carbon number) with moisture levels from 1.0 × 10^2^﻿ to 4.70 × 10^3^ ppm. Acetone and butanone are not included due to convolved peaks for protonated monomer and proton bound dimer. Nonanone was removed due to anomalous patterns necessitating further, detailed study.

The response to ketones at 7 ppb to moisture levels of 1.0 × 10^2^ to 6.00 × 10^3^ ppm is shown in Fig. 12 where the patterns described above for protonated monomer and proton bound dimer are consistent for ketones above hexanone. The pattern of response is progressive loss of proton bound dimer and then protonated monomer until only reactant ion remains above the different moisture level thresholds shown in Table 3. The thresholds, increase proportionally with ketone mass to 5.70 × 10^3^ ppm H_2_O for 2-dodecanone (PA 840 kJ mol^−1^). Findings for acetone and butanone are not included in Fig. 12 due to complete convolution of peaks for protonated monomer and proton bound dimer (see Supplementary Information). Dispersion plots for nonanone exhibited unusual patterns of ion intensity suggestive of ion decomposition. This merits closer inspection perhaps with DMS/Mass Spectrometry but such studies were beyond the scope of this investigation.

At a practical level, individual calibration curves will be needed for each ketone for quantitative monitoring of ketones in ground or surface waters.

At a practical level, individual calibration curves will be needed for each ketone for quantitative monitoring of ketones in ground or surface waters and this technology has demonstrated performance in detection up to 6.00 × 10^3^ ppm with acceptable behavior for ion formation and ion separation by DMS. At a fundamental level of ionization chemistry, levels of moisture entering the ion source should be as low as possible and for best response should not exceed 1.00 × 10^3^ ppm. The technology and operating parameters of this drift tube however necessitate elevated levels of moisture beginning at 1.00 × 10^3^ ppm.

## Conclusions

A DMS analyzer at 30 °C operated over a broad range of moisture levels (1.0 × 10^2^ to 1.71 × 10^4^ ppm) without any anticipated technical problems due to moisture condensation. This study has demonstrated that measurements with a commercial embodiment of DMS at room temperature is possible over a range of water concentration in the gas phase with a conflicting demand of best ionization properties with reduced moisture and stable analyzer performance over 1.00 × 10^3^ ppm. The boundary of analyzer performance is shown to provide acceptable response and chemistry of ion formation. Response was improved by decreasing of moisture level. This is attributed to suppression of ionization as the reactant ion H^+^(H_2_O)_n_ was progressively hydrated and the displacement of H_2_O by M was increasingly disfavored. Although the duty cycle was 5% for the separation waveform, resolution of peaks was achieved and demonstrated the suitability of this instrument for further development as an environmental monitor. Intentions to adapt this analyzer, and other DMS analyzers, for water monitoring should be approached with caution since the influence of moisture levels with relatively high proton affinity ketones portends difficulties under same conditions for substances, of environmental interest with lesser proton affinities. Response to a homologous series of ketones was dependent on molar mass and proton affinities providing a caution that calibrations for individual ketones should be made with DMS in environmental monitoring.

## Supplementary information

Supplementary Dataset 1

## References

[1] Nazarov EG (2012). A journey into DMS/FAIMS technology. International Journal for Ion Mobility Spectrometry.

[2] Kolakowski BM, Mester Z (2007). Review of applications of high-field asymmetric waveform ion mobility spectrometry (FAIMS) and differential mobility spectrometry (DMS). Analyst.

[3] Krylov E, Nazarov EG, Miller RA, Tadjikov B, Eiceman GA (2002). Field dependence of mobilities for gas-phase-protonated monomers and proton-bound dimers of ketones by planar field asymmetric waveform ion mobility spectrometer (PFAIMS). J. Phys. Chem. A.

[4] Krylov EV, Coy SL, Nazarov EG (2009). Temperature effects in differential mobility spectrometry. Int. J. Mass Spectrom..

[5] Krylova N, Krylov E, Eiceman GA, Stone JA (2003). Effect of moisture on the field dependence of mobility for gas-phase ions of organophosphorus compounds at atmospheric pressure with field asymmetric ion mobility spectrometry. J. Phys. Chem. A.

[6] Kuklya A, Uteschil F, Kerpen K, Marks R, Telgheder U (2015). Effect of the humidity on analysis of aromatic compounds with planar differential ion mobility spectrometry. Int. J. Ion Mobil. Spectrom..

[7] Nazarov EG, Coy SL, Krylov EV, Miller RA, Eiceman GA (2006). Pressure effects in differential mobility spectrometry. Anal. Chem..

[8] Shvartsburg AA, Ibrahim YM, Smith RD (2014). Differential ion mobility separations in up to 100% helium using microchips. J. Am. Soc. Mass Spectrom..

[9] Limero, T., Cheng, P., Reese, E., Jones, J. & Wallace, W. Operational Air Quality Monitor: Scientific Studies in Preparation for Flight. In *41st International Conference on Environmental Systems* 77058–77058, 10.2514/6.2011-5024 (2011).

[10] Schneider BB, Covey TR, Coy SL, Krylov EV, Nazarov EG (2010). Planar differential mobility spectrometer as a pre-filter for atmospheric pressure ionization mass spectrometry. Int J Mass Spectrom.

[11] Siegel, M. W. Atmospheric Pressure Ionization. In Plasma *Chromatograph* (ed. Carr, T. W.) 96–113 (Plenum Press, 1984).

[12] Miller RA, Nazarov EG, Eiceman GA, King AT (2001). A MEMS radio-frequency ion mobility spectrometer for chemical vapor detection. Sensors Actuators, A Phys..

[13] Nazarov EG, Miller RA, Eiceman GA, Stone JA (2006). Miniature differential mobility spectrometry using atmospheric pressure photoionization. Anal. Chem..

[14] Wright, J. A., Miller, R. A. & Nazarov, E. G. Atmospheric Pressure Air microplasma Ionization Source for Chemical Analysis Applications. *19th IEEE Int. Conf. Micro Electro Mech. Syst*. 378–381, 10.1109/MEMSYS.2006.1627815 (2006).

[15] Eiceman GA, Krylov EV, Krylova NS, Nazarov EG, Miller RA (2004). Separation of ions from explosives in differential mobility spectrometry by vapor-modified drift gas. Anal. Chem..

[16] Schneider BB, Nazarov EG, Covey TR (2012). Peak capacity in differential mobility spectrometry: Effects of transport gas and gas modifiers. *Int*. J. Ion Mobil. Spectrom..

[17] Rorrer Iii LC, Yost RA (2015). Solvent vapor effects in planar high-field asymmetric waveform ion mobility spectrometry: Solvent trends and temperature effects. Int. J. Mass Spectrom..

[18] Przybylko ARM (1995). The determination of aqueous ammonia by ion mobility spectrometry. Anal. Chim. Acta.

[19] Gabryelski W, Wu F, Froese KL (2003). Comparison of high-field asymmetric waveform ion mobility spectrometry with GC methods in analysis of haloacetic acids in drinking water. Anal. Chem..

[20] Lyczko J, Beach D, Gabryelski W (2015). Detection, Identification, and Occurrence of Thiotetronic Acids in Drinking Water from Underground Sources by Electrospray Ionization-High Field Asymmetric Waveform Ion Mobility Spectrometry-Quadrupole Time-of-Flight-Mass Spectrometry. Anal. Chem..

[21] Márquez-Sillero I, Aguilera-Herrador E, Cárdenas S, Valcárcel M (2011). Ion-mobility spectrometry for environmental analysis. TrAC - Trends Anal. Chem..

[22] Kerpen, K., Kuklya, A., Marks, R., Uteschil, F. & Telgheder, U. Development of an ESI-FAIMS/DMS System for Rapid Water Analysis. in *5th Water Contamination Emergencies: managing the threats* 365–373, 10.1039/9781849737890-00365 (2013).

[23] Rainsberg MR, de Harrington PB (2005). Thermal desorption solid-phase microextraction inlet for differential mobility spectrometry. Appl. Spectrosc..

[24] Holopainen S, Nousiainen M, Sillanpää MET, Anttalainen O (2012). Sample-extraction methods for ion-mobility spectrometry in water analysis. TrAC - Trends Anal. Chem..

[25] Mäkinen M (2010). The effect of humidity on sensitivity of amine detection in ion mobility spectrometry. Talanta.

[26] Sunner J, Nicol G, Kebarle P (1988). Factors Determining Relative Sensitivity of Analytes in Positive. Anal. Chem..

[27] Willy, T. J. Influence of Moisture on the Quantitative Response for Five Chemicals Families in Ion Mobility Spectrometry, M.S. Thesis, New Mexico State University, Las Cruces, NM Dec. (2016).

[28] Borsdorf H, Fiedler P, Mayer T (2015). The effect of humidity on gas sensing with ion mobility spectrometry. Sensors Actuators, B Chem..

[29] Carroll DI, Dzidic I, Stillwell RN (1975). and E. C. H. I. Identification of Positive Reactant ions Observed for Nitrogen Carrier Gas in Plasma Chromatograph Mobility. Studies D. Anal. Chem..

[30] Kim SH, Betty KR, Karasek FW (1978). Mobility behavior and composition of hydrated positive reactant ions in plasma chromatography with nitrogen carrier gas. Anal. Chem..

[31] Anttalainen O (2018). Differential mobility spectrometers with tuneable separation voltage – theoretical models and experimental findings. Trends Anal. Chem..

[32] Schneider, B. B., Covey, T. R. & Nazarov, E. G. DMS-MS separations with different transport gas modifiers, 10.1007/s12127-013-0130-8.

[33] Kebarle P, Searles SK, Zolla A, Scarborough J, Arshadi M (1997). The solvation of the hydrogen ion by water molecules in the gas phase. Heats and entropies of solvation of individual reactions: H + (H2O)(n-1) + H2O → H + (H2O)(n). J. Mass Spectrom..

[34] Meot-Ner, M. The Ionic Hydrogen Bond and Ion Solvation. 1. NH+ O, NH+ N, and OH+···O Bonds. Correlations with Proton Affinity. Deviations due to Structural Effects. *J. Am. Chem. Soc* **106** (1984).

[35] Davidson WR, Sunner J, Kebarle P (1979). Hydrogen Bonding of Water to Onium Ions. Hydration of Substituted pyridinium Ions and Related Systems. J. Am. Chem. Soc..

[36] Acree, W. E., Chickos, Jr. J. S., Linstrom, E. P. J. & Mallard, W. G. Phase Transition Enthalpy Measurements of Organic andOrganometallic Compounds. *NIST Chem. WebBook, NIST Stand. Ref. Database Number 69* 20899, 10.18434/T4D303 (2018).

