# Supplementary material for: Differential Mobility Spectrometry of Ketones in Air at Extreme Levels of Moisture
Source: Sci Rep. 2019 Apr 3;9:5593. doi: 10.1038/s41598-019-41485-7 (PMC6447537; doi:10.1038/s41598-019-41485-7)
Supplement: Supplementary file 1 — Supplementary Dataset 1 [file 41598_2019_41485_MOESM1_ESM.docx]

**Supplementary Information**

**Differential Mobility Spectrometry of Ketones in Air at Extreme Levels of Moisture**

Z. Safaei^1^, M. Sillanpää^1^, G.A. Eiceman^2^, J. Puton^3^, J.A. Stone^4^, M. Nasirikheirabadi^1^, O. Anttalainen^5^,

^1^Department of Green Chemistry, LUT University, Sammonkatu 12, FI-50130 Mikkeli, Finland.

^2^Department of Chemistry and Biochemistry, 1175 North Horseshoe Drive, New Mexico State University, Las Cruces, NM 88003

^3^Institute of Chemistry, Military University of Technology, Kaliskiego 2, Warsaw, Poland

^4^Department of Chemistry, Queens University, Kingston, Ont. K7L 4J1, Canada

^5^Environics Oy, Sammonkatu 12, FI-50130 Mikkeli, Finland

**Acetone at fixed concentration of 400 ppb and 3 moisture levels of 1.0 x 10^2^, 4.1 x 10^2^, and 1.10 x 10^3^ ppm**

Figure 1S. Dispersion plots for acetone at 400 ppb vapor concentration and 1.0 x 10^2^ ppm moisture (B) and for the reactant ion peak as a control measurement (A).

Figure 2S. Dispersion plots for acetone at 400 ppb vapor concentration and 4.1 x 10^2^ ppm moisture (B) and for the reactant ion peak as a control measurement (A).

Figure 3S. Dispersion plots for acetone at 400 ppb vapor concentration and 1.10 x 10^3^ ppm moisture (B) and for the reactant ion peak as a control measurement (A).

Figure 4S. Composition of DMS spectra obtained at 500 V separation voltage at 400 ppb acetone and several moisture levels (see inset).

**2-Butanone at fix concentration of 20 ppb and 3 moisture levels: 1.0 x 10^2^, 5.5 x 10^2^, and 1.25 x 10^3^ ppm**

Figure 5S. Dispersion plots for 2-butanone at 20 ppb vapor concentration and 1.0 x 10^2^ ppm moisture (B) and for the reactant ion peak as a control measurement (A).

Figure 6S. Dispersion plots for 2-butanone at 20 ppb vapor concentration and 5.5 x 10^2^ ppm moisture (B) and for the reactant ion peak as a control measurement (A).

Figure 7S. Dispersion plots for 2-butanone at 20 ppb vapor concentration and 1.25 x 10^3^ ppm moisture (B) and for the reactant ion peak as a control measurement (A).

Figure 8. Composition of DMS spectra obtained at 500 V separation voltage at 400 ppb 2-butanone and several moisture levels (see inset).
